# Estimation of the age of human bloodstains under the simulated indoor and outdoor crime scene conditions by ATR-FTIR spectroscopy

**DOI:** 10.1038/s41598-017-13725-1

**Published:** 2017-10-16

**Authors:** Hancheng Lin, Yinming Zhang, Qi Wang, Bing Li, Ping Huang, Zhenyuan Wang

**Affiliations:** 1 Department of Forensic Pathology, Xi’an Jiaotong University, Xi’an, 710061 P. R. China; 2 Department of Forensic Pathology, Institute of Forensic Science, Ministry of Justice, Shanghai, 200063 P. R. China

## Abstract

Estimation of the age of human bloodstains is of great importance in forensic practices, but it is a challenging task because of the lack of a well-accepted, reliable, and established method. Here, the attenuated total reflection (ATR)-Fourier transform infrared (FTIR) technique combined with advanced chemometric methods was utilized to determine the age of indoor and outdoor bloodstains up to 107 days. The bloodstain storage conditions mimicked crime scene scenarios as closely as possible. Two partial least squares regression models—indoor and outdoor models with 7–85 days—exhibited good performance for external validation, with low values of predictive root mean squared error (5.83 and 4.77) and high R^2^ values (0.94 and 0.96) and residual predictive deviation (4.08 and 5.14), respectively. Two partial least squares–discriminant analysis classification models were built and demonstrated excellent distinction between fresh (age ≤1 d) and older (age >1 d) bloodstains, which is highly valuable for forensic investigations. These findings demonstrate that ATR-FTIR spectroscopy coupled with advanced chemometric methods can be employed as a rapid and non-destructive tool for age estimation of bloodstains in real-world forensic investigation.

## Introduction

Bloodstain is one of the most frequently encountered biological evidences at crime scenes^1^, especially those for violent crimes. Accurate estimation of the age of bloodstains can be a tremendous help for forensic investigators in the reconstruction of the event timeline, determination of the time of death or injury of the victim, and reduction of the pool of suspects^2^.

Numerous methods have been investigated to determine the age of bloodstains, such as use of oxygen electrodes^3^, electron paramagnetic resonance (EPR)^4^, high-performance liquid chromatography (HPLC)^5^ and RNA degradation^6,7^. Unfortunately, these approaches are not robust, limiting their forensic application. In recent years, Agudelo *et al*.^8,9^ investigated human serum for determining the age of bloodstains using bioaffinity-based and biocatalytic assays. However, this method was inappropriate for forensic practice because it in actual cases it is difficult to extract an adequate amount of serum from dried bloodstains. Additionally, several novel techniques have been explored to determine the age of bloodstains, including fluorescence lifetime measurements^10,11^, atomic force microscopy^12^ and the use of smartphones for quantifiable colour change correlations^13^.

Vibrational spectroscopy techniques are becoming more and more popular in forensic science because of their non-destructive, rapid, quantitative, and confirmatory features^14^. The results of several noteworthy studies (2011 and later) investigating the use of spectroscopic methods to estimate bloodstain age are summarized in Table 1. These methods showed an ability to determine bloodstain age, especially when combined with chemometric methods. However, most of the studies were conducted using simulated bloodstain samples under ideal laboratory conditions. In real-world case work, varying ambient conditions will affect the process of bloodstain denaturation and aggregation and ultimately contribute to the complexity and difficulty of bloodstain age estimation. In the present work, an approach combining ATR-FTIR spectroscopy with chemometric methods was established for determining the age of bloodstains up to 107 days. The bloodstain samples were created and stored in indoor and outdoor environments, and the storage conditions closely simulated real crime scenes.

**Table 1:** Summary of spectroscopy techniques proposed for the age determination of bloodstains.

Year	Methods	Storage environment	Chemometrics	Range of age	Error of age prediction	Ref
2011	Reflectance spectroscopy	laboratory conditions	—	0–60 d	—	38
2011	Reflectance spectroscopy	laboratory conditions	LDA	1–19 d	±0.71 d	46
2012	Hyperspectral imaging	simulated crime scene	—	0.1–200 d	13.4% of the actual age	39
2012	Near infrared spectroscopy	laboratory conditions	PLSR	0–28 d	8.9% of the actual age	47
2013	Hyperspectral imaging	laboratory conditions	LDA	0–7, 0–30 d	±0.27 and ± 1.17 d, respectively	48
2016	Raman spectroscopy	laboratory conditions	PLSR	1–168 h	±2.19 h	41
2017	Visible reflectance spectroscopy	calorstats	PCA-SVMR	2 h–45 d	±42.79 h	42

Chemometric methods are capable of extracting useful information from complex spectral datasets to yield more comprehensive and accurate results^15^. The development of reliable and robust chemometric framework to handle analytical data has been identified as an important step in the biological spectroscopy analyses^16–18^. In our study, partial least square regression (PLSR) and partial least square -discrimination analysis (PLS-DA) were employed to analyse blood spectral dataset, since these two PLS approaches have experienced a broad acceptance in the spectral analysis with their powerful ability of dealing with noisy and collinear spectral variables. A good example of PLS approaches in clinical applications is outlined in the study by Khoshmanesh *et al*.^19^, in which, the aim was to detect early-stage malaria parasites in infected erythrocytes. In the field of forensic science, these two methods have also been used, such as in the identification of species’ blood^20,21^ and bone^22^, investigation of burned bones^23^, and profiling of cocaine in seizures^24^. In our laboratory, ATR-FTIR in combination with PLSR has proven to be a good tool for the characterization of post-mortem biochemical changes in rabbit plasma^25,26^. However, one main problem of PLS approaches is over-fitting. To tackle this problem in our study, the constructed PLSR and PLS-DA models based on the resulting spectral dataset were validated with two independent sample datasets, both of which originated from two volunteers whose samples were separate from those used to develop the models.

## Materials and Methods

### Sample preparation

This study was conducted in accordance with the guidelines of the National Institute of Health, China. The protocol was approved by the Ethical Committee of Xi’an Jiaotong University and informed written consent was obtained from all blood donors. Fresh whole-blood samples (without anticoagulants) were obtained from four healthy volunteers (two males and two females) and deposited immediately onto glass slides to form bloodstains. Nineteen time points were set: 0.25, 1, 2, 3, 4, 5, 6, 7, 9, 12, 15, 19, 24, 30, 40, 50, 65, 85, and 107 d. For each time point, 6 bloodstain samples per donor were prepared, of which 3 samples were stored in an indoor environment and 3 in an outdoor environment. It should be emphasized that the indoor condition was not specifically controlled and the samples were exposed to dim sunlight during the day and no light at night. Bloodstain samples placed in the outdoor environment were exposed to the light, heat, and humidity of the outdoor environment but not rain. A total of 556 bloodstain samples were ultimately collected, encompassing an indoor training group of 228 samples and an outdoor training group of 228 samples. These two groups were used for chemometric model constructions. Additionally, according to the aforementioned method, two bloodstain groups (indoor and outdoor groups; each group contained 114 samples with bloodstain age ranging from 0.25 to 107 d) from two other healthy individuals (one male and one female) were prepared. These two groups, called test groups, were employed to validate the constructed chemometric models.

### Spectra collection and data preprocessing

Spectral acquisition was performed using a Nicolet iS 50 FTIR Spectrometer (Thermo Fisher Scientific, Waltham, WA, USA) equipped with an ATR accessory (Thermo Fisher Scientific, Waltham, WA, USA) containing a diamond crystal face approximately 2 mm in diameter. Before each measurement, the bloodstain sample was collected in an Eppendorf tube and mixed with 10 µL of normal saline uniformly. Subsequently, 1 µL of sample was deposited on the ATR crystal face and dried with an air dryer for approximately 4 min. The spectra were recorded in the range of 900–1800 cm^−1^ at a resolution of 4 cm^−1^ with 32 scans. The background spectra were subtracted automatically from the sample spectra. For each sample, 3 replicated spectra were collected and then averaged to form a single spectrum. The spectra were recorded with OMNIC software version 9.2 (Thermo Fisher Scientific, Waltham, WA, USA).

Next, baseline correction, unit vector normalization and multiplicative scatter correction (MSC) preprocessing methods were applied to the 1800-900 cm^−1^ region to eliminate baseline offsets, remove artefacts related to the analytical techniques and samples under study, and reduce the effects of light scattering^27^. The data preprocessing was carried out by Unscrambler 9.7 (CAMO software, Oslo, Norway). The original and pre-processed raw spectra are shown in the Supporting Information.

### Multivariable statistical analysis

PLSR is a multivariate regression method that can decompose the X-variable with the guidance of the Y-variable and find latent variables (LVs), which are linear combinations of the original variables to maximize the co-variation between X and Y during regression^28^. In this work, the X-variable corresponded to the matrix of spectral intensity and the responding Y-variable was associated with age values. PLS-DA is a classification method based on the PLS approach in which the Y-variable is chosen to represent the class membership^29^. PLSR and PLS-DA were established using Matlab software version R2014a (MathWorks, Natick, MA, USA) equipped with PLS Toolbox 8.1.1 (Eigenvector Research, Manson, WA, USA).

To evaluate the stability and predictive ability of PLSR and PLS-DA models, both internal cross-validation (CV) and external validation were performed^30^. In our study, the CV was performed using 10 folds with the Venetian blinds procedure. External validation was performed using constructed models to predict the test bloodstain samples that were stored in the same environment as the training bloodstain samples. Another test, called the “expanding test” (actually, this test is another type of external validation), was performed using constructed models to predict the test samples whose storage environment differed from that of the training samples. The purpose of the expanding test was to explore the predictive power of the models in estimating bloodstain age under multiple environmental conditions.

Root mean square error (RMSE), including calibrated RMSE (RMSEC), cross-validated RMSE (RMSECV) and predicted RMSE (RMSEP), and R^2^ and residual predictive deviation (RPD), as the three main parameters of the model’s calibrated and predicted results, were used to evaluate the regression model reliabilities. High values of R^2^ and RPD and a low value of RMSE demonstrate a well-established PLS regression model. Notably, an RPD value above 3 indicates that the model is very reliable for prediction purposes^31,32^.

### Data availability

All data generated or analysed during this study are available from the corresponding author on reasonable request.

## Results and Discussion

In this work, the studied spectral range of 1800-900 cm^−1^, also called the “biofingerprint region”, offers the most information on the chemical compounds of biological samples^33^, including lipid esters (1800–1700 cm^−1^)^34^; amide I, II and III proteins (1700–1500 cm^−1^, 1350–1200 cm^−1^)^34,35^; and nucleic acids and carbohydrates (1200–900 cm^−1^)^36^. As for bloodstains, the corresponding infrared spectra provide detailed information regarding haemoglobin, which makes up 97% of the dry content of blood^2^.

**Figure 1:**
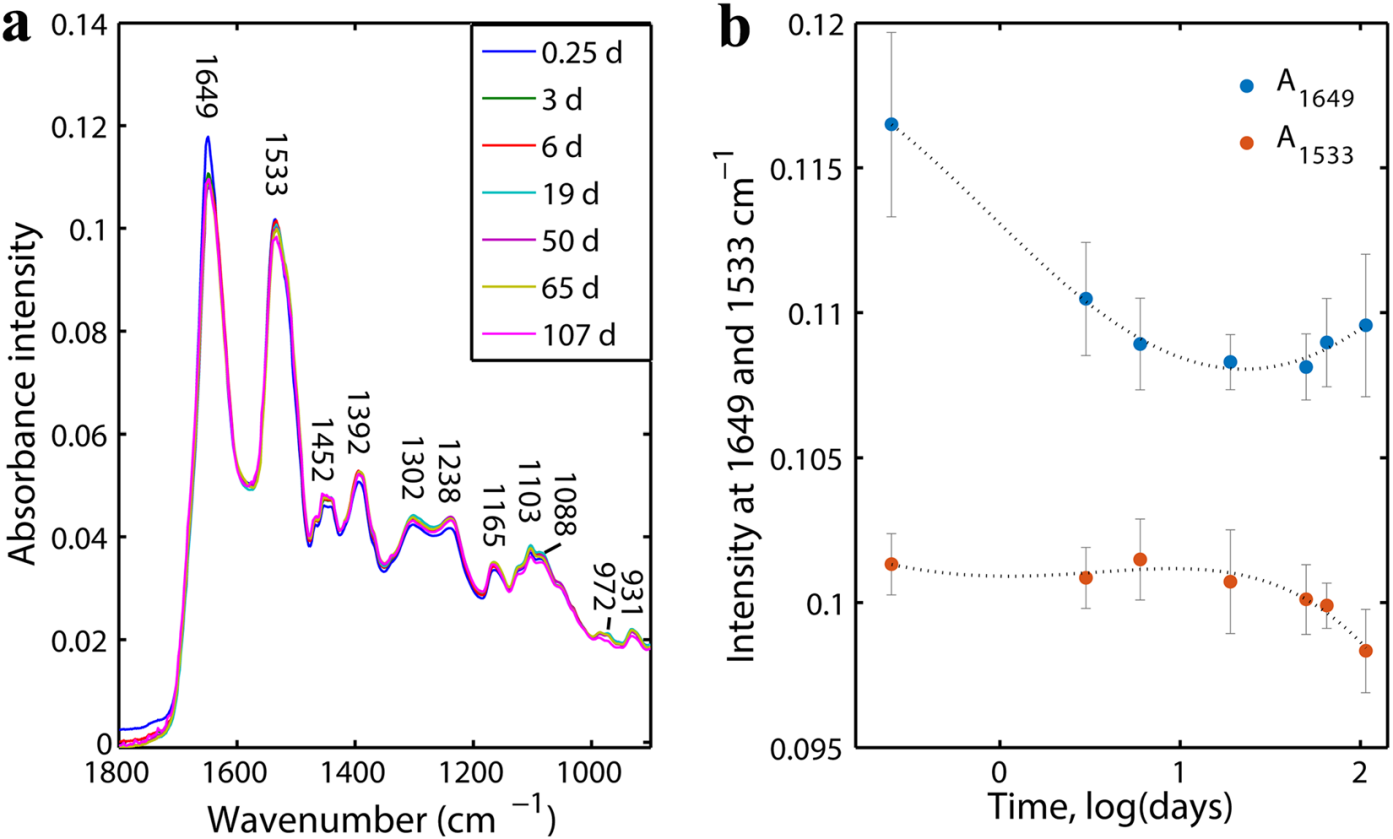
(**a**) FTIR averaged spectra of outdoor bloodstains at different time points in the range of 1800-900 cm^−1^. (**b**) The trends of the intensities of the peaks at 1649 and 1533 cm^−1^ for all spectra over time with polynomial curve fit lines (model = 4).

Figure 1a shows a comparison of the average spectra for the outdoor bloodstains with seven selected ages. The assignments of the main observed bands are tabulated in Table 2. As can be seen in Fig. 1b, the highly varied vibrational bands were at 1649 (corresponding to the α-helix structures of haemoglobin)^37^ and 1533 cm^−1^ (representing amide II). The average absorbance intensity at 1649 cm^−1^ decreased at first, reached the minimum value at age = 19 d, and increased slowly thereafter. However, the average absorbance intensity at 1533 cm^−1^ did not show a decreasing trend until 19 days after deposition. These findings suggest that the secondary structures of haemoglobin changed constantly as age increased, and the most probable cause was associated with the kinetic efforts of haemoglobin (Hb → HbO_2_ → met-Hb → hemichrome) during bloodstain aging^38–40^. When fresh blood was exposed to air, autoxidation of haemoglobin would occur immediately, followed by denaturation and aggregation as time progressed^41^. These findings also helped to confirm that the process of bloodstain degradation started immediately and can be detected in a few hours and over a longer period of time.

**Table 2:** ATR FT-IR peak component assignment of bloodstains.

Frequency (cm^–1^)	Assignment
~931	Symmetric C-O stretching from carbohydrates
~972	Symmetric C-O stretching from carbohydrates
~1088	Symmetric vibration of PO_2_^−^
~1103	Symmetric C-O stretching from carbohydrates
~1165	C-O vibration
~1238	Asymmetric vibration of PO_2_^−^
~1302	Amide III band
~1392	Symmetric vibration of COO^−^ of fatty acids and polysaccharides
~1452	C-H bending from CH_3_
~1533	Amide II band
~1649	α-Helical structures of proteins, amide I

Our results also demonstrated the capacity of the ATR-FTIR technique for detecting changes in minor components such as blood glucose (probable bands around 931, 972, and 1103 cm^−1^), except the dominant spectral changes of haemoglobin and its derivative components of aging bloodstains. However, it is impracticable to estimate the age of a bloodstain with the selection of one or several absorption peaks by visualizing intensity changes because of the overlapping spectral features of bloodstain samples. Hence, in our next step, multivariate chemometric methods, which are powerful in the extraction and analyses of information-rich spectroscopic signals, were utilized to construct the model for bloodstain age estimation.

### Age delimitation by the PLSR model

**Figure 2:**
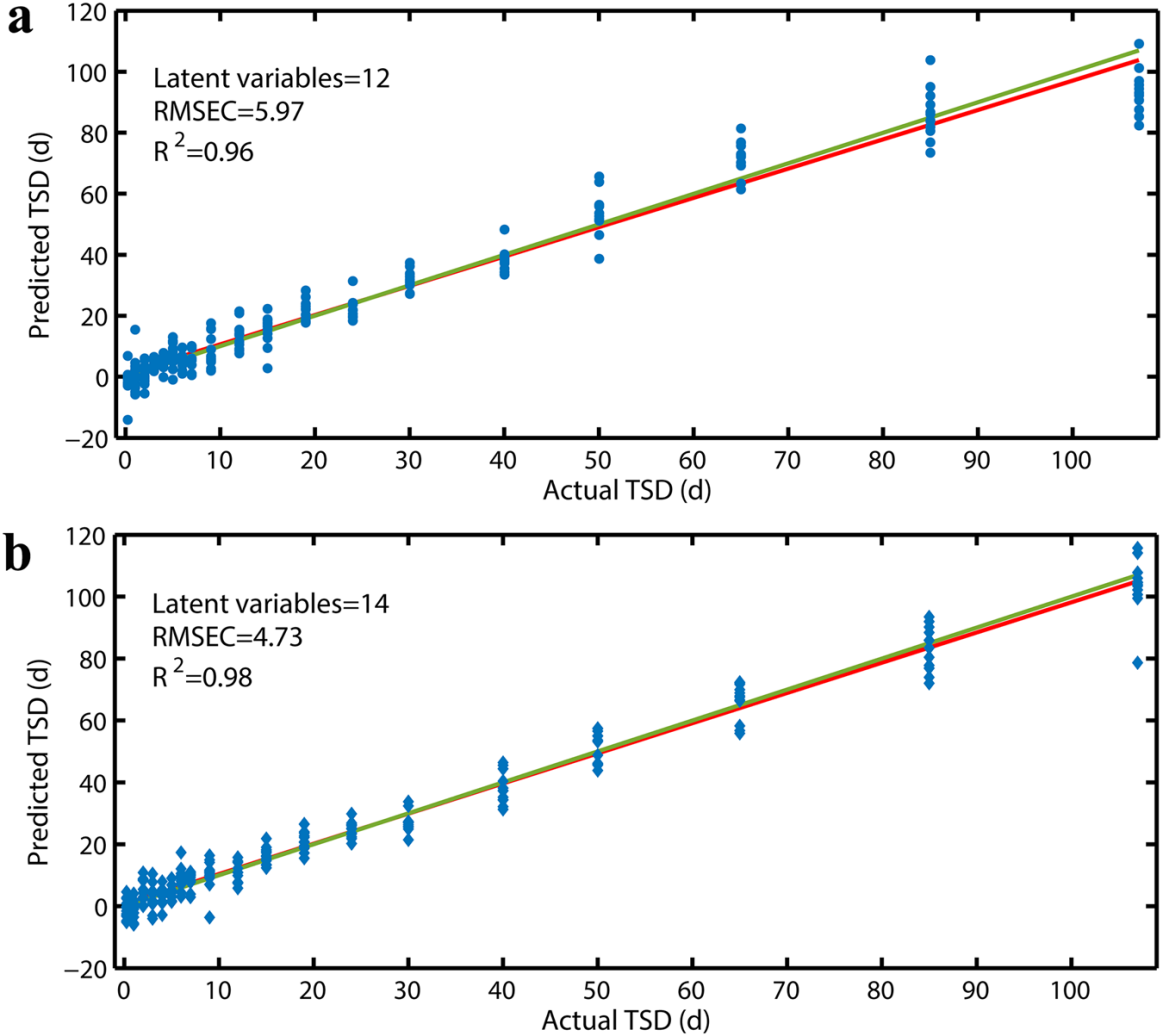
PLSR plots for (**a**) indoor and (**b**) outdoor bloodstain samples in the 0.25- to 107-d period showing the calibrated age predictions versus the actual age.

PLS regression analysis was performed with 12 and 14 LVs, to deliver satisfactory prediction performances and to build models for age estimation of indoor and outdoor bloodstains over the entire age period (0.25–107 d). Figure 2a and b illustrate the calibration results of the indoor and outdoor PLSR models, both of which exhibited good predictive ability as reflected by the R^2^ of 0.96 and 0.98 and RMSEC of 5.97 and 4.73 d, respectively.

Internal cross-validation, as a routine method to determine the number of LVs, was also employed to evaluate the robustness of the calibration model. A stable prediction PLSR model is expected to have a high R^2^ value and a low RMSECV value while achieving prediction performance comparable to that of the calibrated model.

The cross-validated results are presented in Table 3. The R^2^ and RMSECV values were 0.94 and 7.51, respectively, for the indoor regression model and 0.96 and 6.31 for the outdoor regression model. Although the RMSECV values were slightly higher than those of RMSEC, the comparable values of R^2^ demonstrated a good overall fit for the internal cross-validations; thus, our two PLSR models can be considered robust and reliable.

**Table 3:** The validation results of PLSR models in the three time periods.

Time since deposition	Cross-validation		External validation			Expanding test		
	RMSECV	R^2^	RMSEP	R^2^	RPD	RMSEP	R^2^	RPD
Indoor model								
0.25–7 d	1.20	0.72	1.18	0.72	1.90	1.53	0.53	1.19
7–85 d	5.88	0.94	5.83	0.94	4.08	12.93	0.71	1.73
0.25–107 d	7.51	0.94	7.24	0.94	4.20	13.34	0.80	2.24
Outdoor model								
0.25–7 d	0.91	0.84	1.10	0.76	2.09	2.15	0.08	0.99
7–85 d	6.35	0.93	4.77	0.96	5.14	19.99	0.31	0.70
0.25–107 d	6.31	0.96	6.43	0.95	4.42	23.61	0.38	0.77

Due to the limited scale of training bloodstains and high intra-species biodiversity, external validation was subsequently conducted to assess the models’ predictive power using bloodstain samples from two other donors (these donors were outside the training dataset). The external validation performances of the two PLSR models are summarized in Table 3. The high values of RPD and similar values of RMSE and R^2^ of the external validations compared to those of the cross-validations indicated that our PLSR models could be considered very reliable for estimating bloodstain age under the same environmental conditions. Expanding tests were also performed to assess how well our established PLSR models predicted the age of bloodstains under multiple environmental conditions. However, the higher RMSEP values and poorer values of RPD and R^2^ (see Table 3) indicated the unreliability of the established models to predict bloodstain age under various environmental conditions.

In the ideal linear regression with regard to Fig. 2, all spectra (symbols) should lie directly on the line of best fit (the green line), and the minimal spread should be within the symbols for each age point. However, it was observed by visualization that the bloodstain spectra in the “fresh” time period (0.25-7 d in indoor and outdoor PLSR models) exhibited relatively larger discrepancies than the spectral points at age onwards. In particular, the discrepancies were even larger at the 0.25-d and 1-d time points in the indoor PLSR model. It was also observed that the spectral points at 107 d were almost off the fitting line in the indoor PLSR model. This result indicated that the indoor PLSR model was not appropriate for estimating the age of bloodstains that were approaching 107 days old.

**Figure 3:**
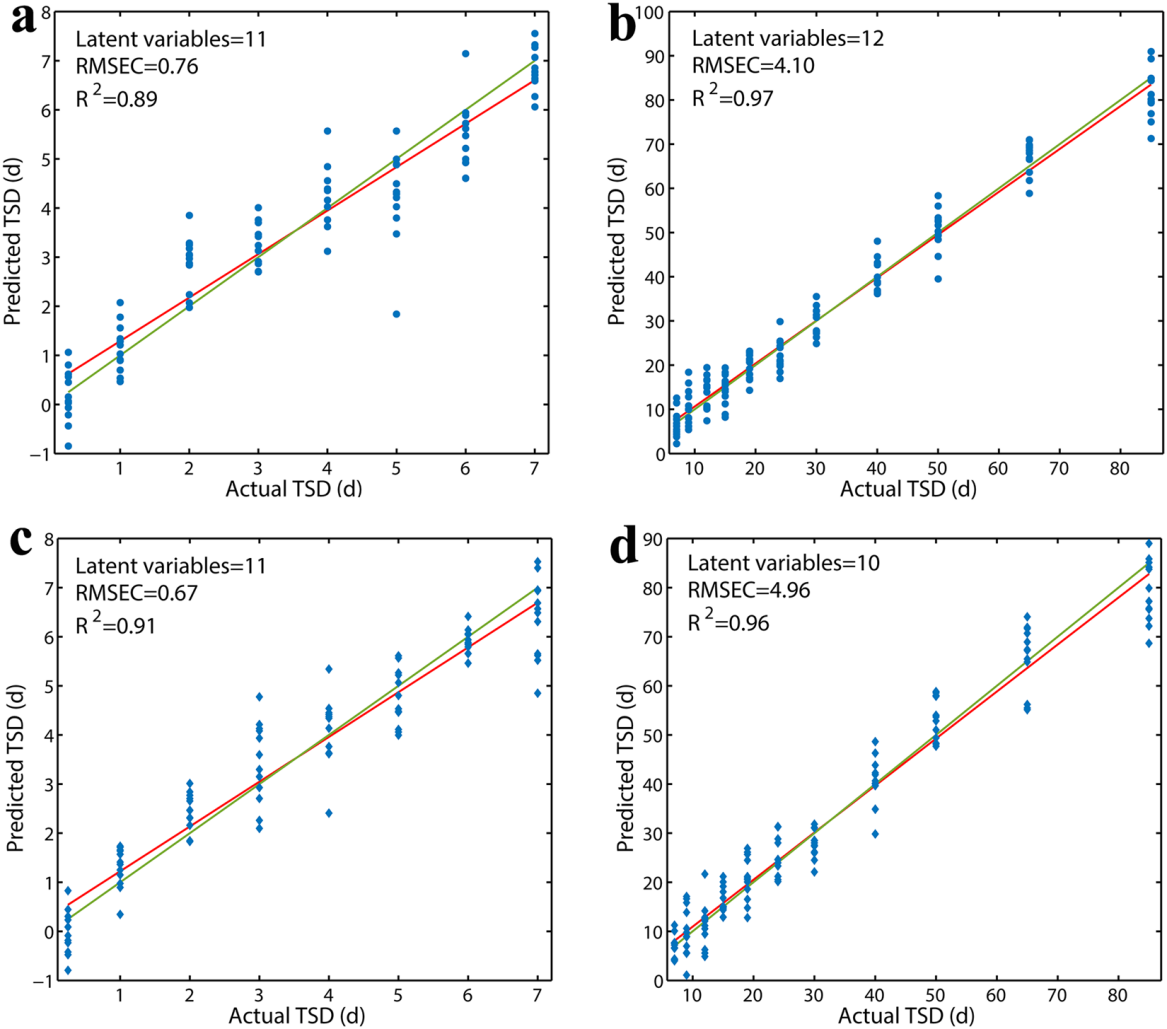
PLSR plots for indoor bloodstain samples in the (**a**) 0.25- to 7-d and (**b**) 7- to 85-d periods and outdoor bloodstain samples in the (**c**) 0.25- to 7-d and (**d**) 7- to 85-d periods showing the calibrated age predictions versus the actual age.

Given the great forensic importance of timely estimation of bloodstain age and the PLSR models’ limited capacity for estimating the age of older bloodstains, in the next step we reconstructed indoor and outdoor PLSR models with split age periods (one age period from 0.25 to 7 days and one from 7 to 85 days before performing the external validation and expanding tests. The age period of the test samples was consistent with that of the training samples for calibration in this study. The calibrated and validated results of the models are presented in Fig. 3 and Table 3, respectively. As can be observed, the calibration and internal cross-validation statistical parameters for the outdoor model in the 7-85-d time period were slightly lower than those for the outdoor model in the entire time period. Nevertheless, the much better validated results—higher RPD value (5.14) and lower RMSEP value (4.77)—and the smaller number of LVs (10) demonstrated that our model was simple, robust and very reliable for prediction purposes. Better performance was also achieved with the indoor model in the 7- to 85-d time period; lower values of RMSEC (4.96), RMSECV (5.88), and RMSEP (5.83) indicated a higher accuracy of age predictions.

Our study also revealed that the indoor and outdoor PLSR models in the 0.25- to 7-d period were not appropriate for prediction of bloodstain age as reflected by the lower values of RPD (1.90 and 2.09, respectively). This apparent uncertainty in the early stage of the models was similar to that with the results published by Sun *et al*.^42^, who employed visible reflectance spectroscopy coupled with SVM to determine bloodstain age.

A possible explanation for the early-stage uncertainty is associated with the reaction kinetics of haemoglobin. According to the results obtained by Tsuruga *et al*.^43^, the autoxidation process of HbO_2_ can be divided into an initial fast decay and final slow decay. In one study by Bremmer *et al*.^40^, the initial fast decay lasted a few hours and then transited to the slow decay. In another study by Bremmer *et al*.^38^, the slow decay probably lasted ten days and entered into a slower decay phase subsequently. Additionally, the study results of the Bremmer research group demonstrated that oxidation rates of HbO_2_ are strongly temperature-dependent and that the transition of met-Hb into hemichrome is strongly humidity-dependent^40^. It is conceivable that the fluctuating temperature and humidity in both the indoor and outdoor environments where the bloodstains were stored resulted in the instability of the autoxidation process of HbO_2_ and increased the complexity of the haemoglobin reaction kinetics in the 0.25-7-d period, which was probably corresponding to the early phase of slow decay. As a consequence, the variety and relative quantity of secondary structures of haemoglobin and its derivatives changed rapidly and irregularly, which in turn led to a relatively trendless variation of the corresponding spectral features (mainly amide regions; see Fig. 1) and resulted in a relatively large variation in the age prediction of bloodstains in the early time period (0.25-7 d). Additionally, the unreliability of all four reconstructed models (presented in Table 3), as well as that of the two previous PLSR models, in predicting bloodstain age under different environments in the entire time period showed that the contribution of environmental factors to the degradation of bloodstains was large.

### Distinguishing between fresh and older bloodstains via PLS-DA

**Figure 4:**
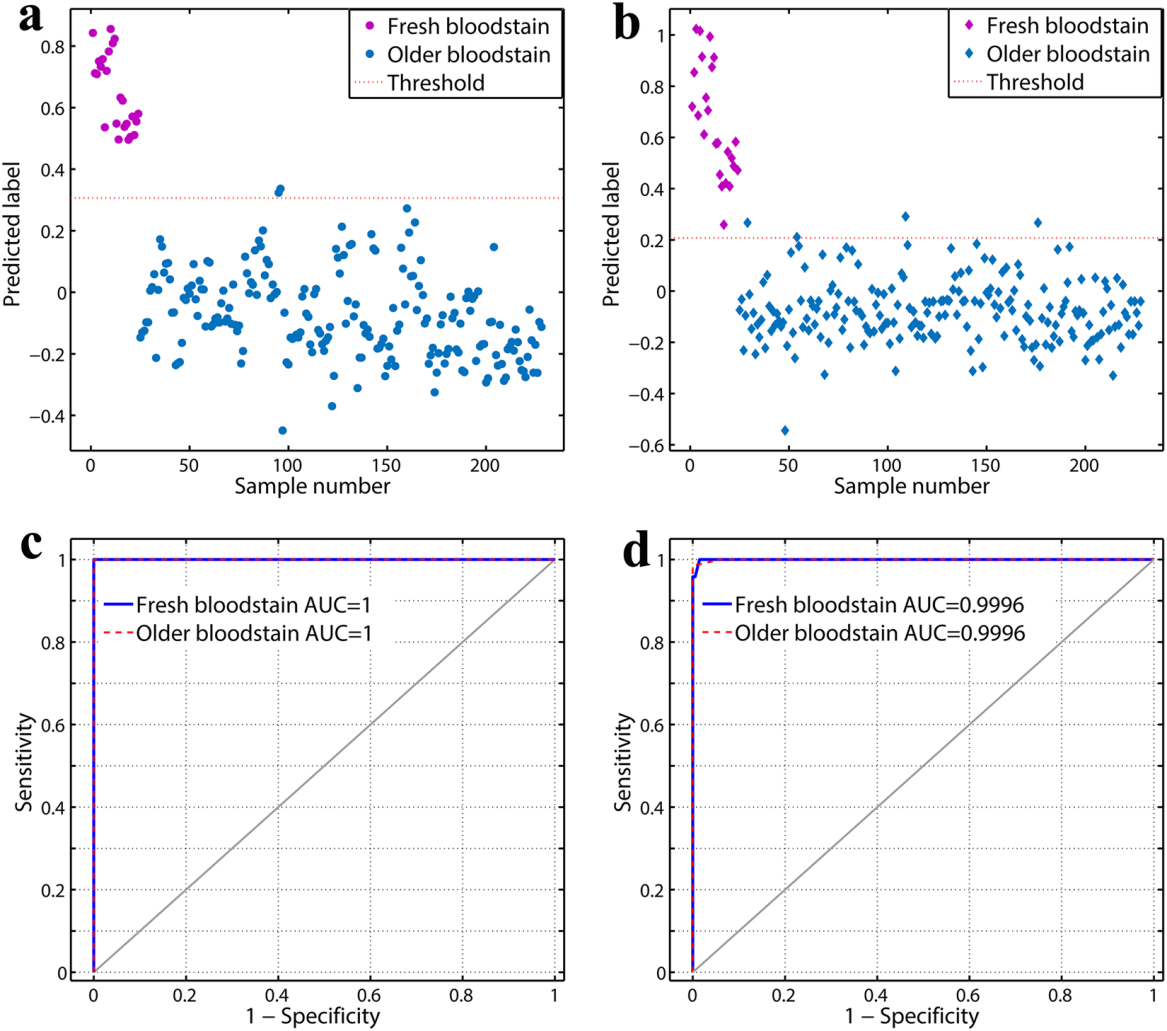
(**a**) Prediction scores of the indoor training dataset using the indoor PLS-DA model. (**b**) Prediction scores of the outdoor training dataset using the outdoor PLS-DA model. The red dotted line represents the default classification threshold. ROC curves with AUC for fresh and older bloodstain classes in the (**c**) indoor and (**d**) outdoor PLS-DA classification models. Random choice is denoted by the grey diagonal line.

Two binary PLS-DA classification models (indoor and outdoor models) were developed using spectra originating from 228 indoor training samples and 228 outdoor training samples. Each spectrum was classified as either a fresh (age ≤ 1 d) or older (age > 1 d) bloodstain. The models were built with 8 and 9 LVs, respectively—the minimum numbers of LVs that delivered satisfactory classification. As seen from Fig. 4a and b, both models demonstrated good separation between these two classes. All the spectra belonging to the fresh bloodstains were classified as the fresh-bloodstain class, and spectra for only two indoor older bloodstains and four outdoor older bloodstains were misclassified in their respective model. The accuracies of the models were 0.99 and 0.98, respectively.

Receiver operating characteristic (ROC) analyses^44^ were conducted to evaluate the discriminatory power of our PLS-DA classification models. The ROC curve was plotted as a function of sensitivity versus 1-specificity (see Fig. 4c and d). The area under the ROC curve (AUC)^44^ was calculated to assess how well the classification model divided the samples to the positive class. AUC has values in the interval [0, 1], where a value of 0.5 means a random classification and 1 means perfect performance. For our two models developed to differentiate between fresh and older bloodstains under indoor and outdoor environments, the AUC values of ROC curves were 1 and 0.9996, respectively, which confirmed the classification capabilities of the models. Validations were also performed to evaluate the reliability and classification ability of the classification models in three different manners. The validated results are summarized in Table 4.

**Table 4:** PLS-DA classification parameters obtained in the cross-validation, external validation and expanding tests.

	Accuracy	Fresh bloodstain (age ≤ 1 d)		Older bloodstain (age > 1 d)	
		Sensitivity	Specificity	Sensitivity	Specificity
Indoor model					
Cross-validation	0.99	0.99	1	1	0.99
External validation	0.99	1	0.99	0.99	1
Expanding test	0.92	0.25	1	1	0.25
Outdoor model					
Cross-validation	0.96	0.96	0.96	0.96	0.96
External validation	0.99	1	0.99	0.99	1
Expanding test	0.85	0.92	0.84	0.84	0.92

Classification performances of internal cross-validation and external validation were as perfect as that of the calibrated PLS-DA models. The classification parameters such as accuracy rate, class sensitivity and specificity were close to 1, similar to those obtained in the calibrated models, indicating that these two calibrated PLS-DA models were robust, reliable and well-fitted for classification purposes. The expanding test was used to attest the ability of each PLS-DA model to discriminate between fresh and older bloodstains in different environments. With regard to discriminating fresh bloodstains, the outdoor classification model showed better performance with a sensitivity value of 0.92 (only one fresh bloodstain from the indoor environment was misclassified) compared with the indoor classification model for classifying outdoor fresh bloodstains (the sensitivity value was only 0.25). The characteristics of the PLS-DA classification models, as well as the aforementioned PLSR models, were defined by each LV’s loading variable, which contained numerous peaks throughout the spectral “fingerprint region” (1800-900 cm^−1^). In other words, spectral information related to all chemical components of a bloodstain during aging contributed to the constructions of the models. This was in accordance with the degradation of bloodstains, which incorporates oxidation of haemoglobin, RNA degradation, and degradation of serum proteins of blood plasma and other blood components^2^. Therefore, this is one feature of our approach that can easily probe the spectroscopic statistical differentiation of the chemical components of bloodstain samples without knowing the specific components.

In conclusion, ATR-FTIR spectroscopy is rapid, easy to use, and non-destructive—properties that are favourable in forensic practice. Its application in bloodstain identification and species determination has been reported previously^21,45^. Nevertheless, to the best of our knowledge, this is the first study demonstrating that ATR-FTIR spectroscopy can be a valuable tool for estimating bloodstain age in mimicked indoor and outdoor crime scenes. Chemometric analysis proved to be powerful for extracting and analysing the universal biospectral information of bloodstains with aging and establishing prediction models for age estimation.

Notably, our approach was more useful for longer-term (7-85 d) estimation of the age of bloodstains regardless of whether they were in an indoor or outdoor environment. The rough performance of our PLSR models in predicting the age of bloodstains in the 0.25- to 7-d time period was partly compensated for by two PLS-DA classification models, which could easily discriminate fresh (age ≤ 1 d) bloodstains from older (age > 1 d) bloodstains in both indoor and outdoor environments. This discrimination was a key finding of our study, and it is highly desirable because it can be applied to forensic practices to help reconstruct a more realistic timeline of events. Nevertheless, prior to applying our approach in real forensic practice, more work needs to be done. Expanding the number of donors, determining the effect of common substrates and contaminations, and developing a robust chemometric framework are important tasks for the future studies.

## Electronic supplementary material

Supplementary Information
