# Supplementary material for: Estimation of the age of human bloodstains under the simulated indoor and outdoor crime scene conditions by ATR-FTIR spectroscopy
Source: Sci Rep. 2017 Oct 16;7:13254. doi: 10.1038/s41598-017-13725-1 (PMC5643403; doi:10.1038/s41598-017-13725-1)
Supplement: Supplementary file 1 — Supplementary Information [file 41598_2017_13725_MOESM1_ESM.docx]

**Supplementary Information**

**Estimation of the age of human bloodstains under the simulated indoor and outdoor crime scene conditions by ATR-FTIR spectroscopy**

Hancheng Lin^1, 2^, Yinming Zhang^1^, Qi Wang^1^, Bing Li^1^, Ping Huang^2, *^, and Zhenyuan Wang^1, *^

^1^Department of Forensic Pathology, Xi’an Jiaotong University, Xi’an, 710061, P. R. China

^2^Department of Forensic Pathology, Institute of Forensic Science, Ministry of Justice, Shanghai, 200063, P. R. China

*** Corresponding authors:**

Zhenyuan Wang (email: wzy218@mail.xjtu.edu.cn)

Ping Huang (emails: huangp@ssfjd.cn)


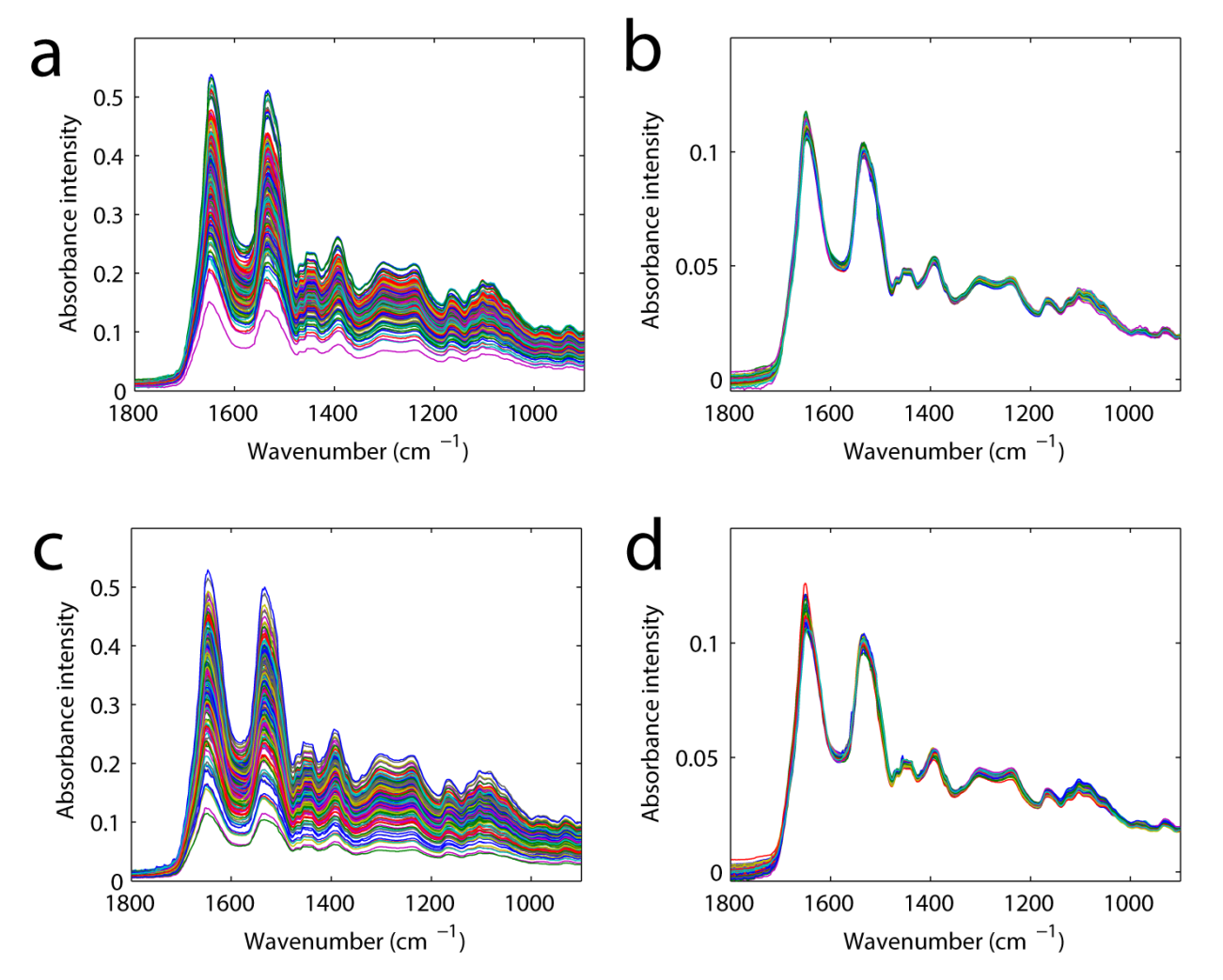


Supplementary Figure S1. The original (a) and pre-processed (b) row spectra for indoor bloodstain samples. The original (c) and pre-processed (d) row spectra for outdoor bloodstain samples.
